# Transportation in the Interstitial Space of the Brain Can Be Regulated by Neuronal Excitation

**DOI:** 10.1038/srep17673

**Published:** 2015-12-03

**Authors:** Chunyan Shi, Yiming Lei, Hongbin Han, Long Zuo, Junhao Yan, Qingyuan He, Lan Yuan, Huipo Liu, Ge Xu, Weiguo Xu

**Affiliations:** 1 Department of Radiology, Peking University Third Hospital, Peking University, Beijing 100191, China; 2 Beijing Key Lab. of Magnetic Resonance Imaging Technology, Beijing 100191, China; 3 School of Electronics Engineering and Computer Science, Peking University, Beijing 100871, China; 4 Department of Anatomy and Histology, School of Basic Medical Sciences, Peking University, Beijing 100191, China; 5 Peking University Medical and Health Analysis Center, Peking University Health Science Center, Beijing 100191, China; 6 Institute of Applied Physics and Computational Mathematics, Beijing 100094, China; 7 Sydney Medical School, Sydney University, Sydney 2006, Australia; 8 National Natural Science Foundation of China, Beijing 100085, China

## Abstract

The transportation of substances in the interstitial space (ISS) is crucial for the maintenance of brain homeostasis, however its link to neuronal activity remains unclear. Here, we report a marked reduction in substance transportation in the ISS after neuronal excitation. Using a tracer-based method, water molecules in the interstitial fluid (ISF) could be specifically visualized in magnetic resonance (MR) imaging. We first observed the flow of ISF in the thalamus and caudate nucleus of a rat. The ISF flow was then modulated using a painful stimulation model. We demonstrated that the flow of ISF slowed significantly following neuronal activity in the thalamus. This reduction in ISF flow continued for hours and was not accompanied by slow diffusion into the ISS. This observation suggests that the transportation of substances into the ISS can be regulated with a selective external stimulation.

The role of the brain interstitial space (ISS) in maintaining the homeostasis of neurons has attracted considerable attention^1^,^2^,^3^. The ISS occupies approximately 20% of the total brain volume and is filled with interstitial fluid (ISF), which contains various ions and organic molecules, including nutrients, waste products, peptides, hormones and neurotransmitters^4^. Given that ISF flow is essential for the transportation of metabolites and nutrients, it is reasonable to assume that neuronal activity is related to the flow rate. The volume of cortical ISS increases by more than 60% during sleep, which may facilitate the clearance of neurodegenerative products that have accumulated during wakefulness^2^. Although a wide range of connections exist between the cerebral cortex, subcortical nuclei, thalamus, brainstem, and basal ganglia, currently it is unclear how either transportation into the ISS or ISF flow responds to the underlying neuronal excitation^2^,^5^. Therefore, *in vivo* global monitoring of the cerebral ISF flow process allows for the investigation of the link between the ISF flow and the excited brain regions.

There are four approaches to measuring the ISS in the living brain: ion-selective microelectrodes (ISMs), microdialysis, integrative optical imaging (IOI), and tracer-based MR imaging^6^,^7^. Although most of the current knowledge of the brain extracellular space in the live animals originates from the results of ISMs^8^,^9^,^10^, microdialysis is the only sampling technique that can continuously monitor the metabolites of the brain^11^,^12^,^13^. However, both ISM and microdialysis techniques can only locally detect the brain ISS. With the aid of fluorescent probes, the IOI method can image the transport of substances of the brain ISS into the cortex to approximately 200 microns in depth^14^,^15^. Thus far, tracer-based MR imaging is the only measurement technique that provides a three-dimensional visualization of the dynamic drainage flow of the brain ISF on a whole-brain scale.

The procedure to visualize and quantify the dynamics of brain ISF in this study is illustrated in Fig. 1a, in which gadolinium-diethylene triamine pentacetic acid (Gd-DTPA) is used to trace the flow of the brain ISF^7^ (Fig. 1). The water-soluble chelate Gd-DTPA is a stable extracellular MR imaging contrast agent^16^. After being introduced into the brain ISS, Gd-DTPA can shorten the spin-lattice relaxation time of hydrogen nuclei in water molecules within a distance range of 2.41–2.5 angstroms^16^. These affected water molecules show a high signal on a T1-weighted MR image (T1WI), and the flow process of the traced brain ISF can be dynamically imaged using a series of MR scans. Due to the diffusion and bulk flow of Gd-DTPA, the enhanced area of the high-intensity signal spreads, thereby resulting in a decrease in high-intensity MR images over time. To calculate the biophysical parameters of the brain ISS, the sequential MR images at various time points are co-registered and the images before injection subtracted. The net signal enhancement can be converted to the tracer’s concentration using a pre-calibrated fitting curve^17^. According to the classical diffusion equation, the diffusion coefficient *D* and clearance coefficient *k’* of the brain ISS can be calculated from the concentration-time profile^18^ (Fig. 1a). Moreover, the flow properties of the traced brain ISF can be quantitatively measured and depicted as *Vd*_max_, time to *Vd*_max_, and half-life (*t*_½_,). Here, *Vd*_max_ is defined as the ratio of the maximum distribution volume of the traced ISF to the total rat brain volume on MR images^7^. As the clearance of the Gd-DTPA fits well by a mono-exponential decay function, *t*_½_ represent the clearance rate or the transportation speed of the tracer in brain ISS.

We selected the thalamus as the region of interest to assess the response of ISF flow to neuronal excitation following painful stimulation (Fig. 1b). The third-stage neurons in the nociceptive afferent pathway are located in the thalamus^19^. The rat forepaw electrical stimulation model that was used in the present study is a well-established model to enhance neuronal activity in the thalamus of conscious rats^20^. To investigate the link between brain ISF flow and neuronal activities, we first observed the ISF flow process in the pain-related thalamus without any external stimulation and compared it with the ISF flow process in the non-pain-related caudate nucleus of the rat brain (Fig. 2). The caudate nucleus mainly modulates voluntary movements and we also recorded neuronal activity in this region during painful stimulation^21^(see details in Fig. 3). Following baseline recording, the ISF flows in the two regions were interrupted by a period of painful electrical stimulation, which was administered at two intensity levels of 3 mA and 5 mA. The time points required to conduct the stimulation are shown in Fig. 1b. Subsequently, the response of the brain ISF flow following neuronal excitation in the thalamus was measured and compared with the response of the non-excited regions in the caudate nucleus.

## Results

### Transport of substances and brain ISF flow processes in thalamus and caudate nucleus without painful stimulation

We demonstrated that the transportation of small water-soluble molecules, such as Gd-DPTA, from the deep center of the brain was not distributed globally but instead flowed in separate divisions with different *Vd*_*max*_ and at different speeds (Fig. 2). The tracer in the brain ISS of the caudate nucleus was distributed more extensively and its final distribution is in a wedge shape with its wide bottom towards the ipsilateral frontotemporal cortex. No tracer flowed in the opposite direction to the thalamic region (Fig. 2A,C,E). *Vd*_*max*_ of the caudate nucleus was found to be 10.27 ± 0.19%, and the time to *Vd*_max_ was 3 hours with a *t*_½_ of 1.46 ± 0.56 hours (*P *<* *0.05). The diffusion coefficient (*D*) in the caudate nucleus was found to be 3.33 ± 0.69×10^−4^  mm^2^/s, with a local clearance rate of 0.79 ± 0.08 × 10^−4^/s.

The flow of the traced ISF in the thalamus was distributed mostly within the anatomical division of the thalamus, and no extension to the caudate nucleus was observed (Fig. 2B,D,F). The time to reach *Vd*_max_ in the thalamus was 2 hours which was faster than that in the caudate nucleus (3 hours) (*P *<* *0.05; Fig. 2G,H). The half-life *t*_½_ of the traced ISF flow in thalamus was 0.81 ± 0.03 hours, significantly shorter than that in the caudate nucleus (1.46 ± 0.56 hours; *P* < 0.05; Fig. 2I). The clearance coefficient *k’* in the thalamus was also larger than that in the caudate nucleus (1.56 ± 0.14 × 10^–4^/s vs 0.79 ± 0.78 × 10^–4^/s, *P* < 0.05; Fig. 2J). The diffusion coefficient *D* in the thalamus was 3.37 ± 0.45×10^–4^  mm^2^/s, similar to that in the caudate nucleus (*P* > 0.05; Fig. 2K).

### Recording the neuronal activities under stimulation

Neuronal sparks in the rat thalamus evoked by the painful stimulation were recorded under anesthesia (Fig. 3A,B) and during an awake state (Fig. 3C,D). We observed no significant differences between the neuronal responses before (Fig. 3A,C) and after (Fig. 3B,D) Gd-DTPA injection. Further, no neuronal activity was recorded in the caudate nucleus following painful stimulation (data not shown).

### Variations in the transport of substances and local brain ISF flow in thalamus and caudate nucleus following painful stimulation

Following painful stimulation, a marked reduction in brain ISF flow was observed in the thalamic region (Fig. 4A–C); however, no change was observed in the caudate nucleus. A comparison of *t*_½_, *k’* and *D* in all groups are shown in Fig. 4. Specifically, *t*_½_ was prolonged to 1.49 ± 0.13 hours in the thalamus with an applied electric current of 3 mA and to 1.50 ± 0.18 hours with 5 mA (Fig. 4D). *k’* was found to be respectively 0.78 ± 0.41 × 10^–4^/s with 3 mA stimulation and 0.76 ± 0.33 × 10^–4^/s with 5 mA stimulation (Fig. 4E). The *k’* values in both cases were smaller than those without painful stimulation (1.55 ± 0.39 × 10^–4^ s, *P* < 0.05). No statistical differences in *k’, t*_½_ and *D* were observed when the stimulation was increased from 3 mA to 5 mA (*P* > 0.05; Fig. 4D–F).

In contrast, no significant change in brain ISF flow in the caudate nucleus was observed following painful stimulation (Fig. 5). Specifically, no statistical differences were observed for *t*_½_ and *D* in the caudate nucleus with and without stimulation (*P* > 0.05). Only a relatively small change in *k’* was found with (0.65 ± 0.11 × 10^–4^/s) and without (0.79 ± 0.21 × 10^–4^/s) stimulation (*P* > 0.05).

## Discussion

In the present study, we found that the transportation of small water-soluble molecules, such as Gd-DPTA, from the deep center of the brain did not have uniform flow in the brain but rather had different *Vd*_max_ values and flow speeds in different regions (Fig. 2). More importantly, we observed a significant slowdown of ISF flow following stimulated neuronal activity in the thalamus region. This phenomenon persisted for several hours and was not accompanied by a slow diffusion of substances. In contrast, transport of substances in the ISS of the brain was not disturbed in the caudate nucleus region following stimulation.

The brain ISS has been hypothesized to be highly connected so that at any given location in the brain extracellular space, molecules such as water could travel through multiple pathways to reach another location^10^. However, we did not observe such a global connectivity in this study. Rather, our results suggest that a new division system could be identified based on the brain ISF flow distribution in different territories. Further, the brain ISS has been widely regarded as an extracellular space that only helps sustain cell viability. However, an increasing number of recent studies have indicated that the brain ISS may have active roles, such as providing a signaling and communication pathway among neural cells, facilitating the coordinated response to cognition, consciousness, emotion and changes in the environment^7^,^22^,^23^,^24^. Our approach enabled the visualization and analysis of the transport of small molecules in the brain ISS at a scale of the whole brain. The marked reduction in the brain ISF flow following neuronal excitation suggests a new mechanism for brain fatigue and sleep requirement: while performing work, more stimuli and subsequent neuronal activities may reduce the local transportation of substances in the brain ISS, followed by the accumulation of waste products and some active substances, such as 5-hydroxytryptamine and dopamine. This increased accumulation is believed to inactivate neuronal excitation and cause fatigue or drowsiness. On the other hand, the relatively faster ISF flow during the resting state may accelerate the local transport of substances and refresh the brain microenvironment.

Determining the variation of ISF flow in different regions of the brain and the location-dependent properties of substance transportation in the ISS will not only improve the understanding of the functional roles of the extracellular space but also provide a useful tool to optimize the techniques for local brain drug delivery^25^,^26^. As discussed in the introduction section, the tracer-based MRI method can dynamically image the global drainage flow of brain ISF. Additionally, the use of Gd-DTPA specifically traces the endogenous water molecules in brain ISF^27^. Thus, the tracer-based MRI method has a special advantage in analyzing and predicting the dynamic distribution of water-soluble drugs in the brain^28^. A new perspective is therefore provided for developing a strategy for delivering drugs in the CNS^7^. First, we verified that drug distribution in the brain could be location dependent and that the transport of drug molecules in different ISS regions could occur at different speeds. Second, we revealed that the distribution and clearance rate in each specific region of the brain is regulated in part by selective external stimulation. Thus, a new pharmacokinetics model is needed for drug distribution in the brain, particularly water-soluble, small-molecular agents that can be distributed throughout the brain ISS.

Substance transport in ISS is a complicated process and is determined by both the biophysical properties of the brain ISS and the chemical properties of the substances in the brain ISF. Recent studies have verified that both bulk flow and diffusion are mechanisms for substance transport in ISS^29^, while a transient slow diffusion has been reported in the activated cortex^5^. It is highly unlikely that alterations to ISS boundary structures, including cell membrane and extracellular matrix is responsible for the down-modulation of brain ISF flow following neuronal stimulation, since the associated potential change in cell membrane and the vascular response are short-term events that last only milliseconds to seconds^30^,^31^,^32^, while the slow ISF flow observed in the current study is long lasting.

Here we propose two potential mechanisms for the down-modulation of brain ISF flow following neuronal stimulation (Fig. 6). The first is a biochemical mechanism that involves the characteristics of the probe or tracer. The ion gadolinium is chelated into a cavity of DTPA, resulting in two residual negative charges^33^. The neuronal excitation is followed by a series of chemical activities, such as ion exchange, nutrient consumption, waste product accumulation and neurotransmitter release^34^,^35^,^36^. The local potential is thereafter affected by the release of charged substances, such as positive ions, glutamate and DA^37^, which impede the movement of the charged tracer. The second is a biomechanical mechanism that involves neuronal excitation due to instantaneous neural cell swelling, especially in astrocytes^38^. During excitation, the swelling of astrocytes is accompanied by a reduction in ECS volume of up to 30%^39^,^40^,^41^. Moreover, the amount of astrocytes in the thalamus is approximately 2–10 times that of neurons^42^,^43^. During excitation, swollen cells will distort adjacent cells in the non-excited area, where the space between the cells will be significantly narrowed or even eliminated, thereby blocking the downstream hydrodynamic path. Thus, the tracer molecules between excited cells may be squeezed out and accumulate in the space between excited and non-excited neurons^44^. After excitation is ended, the flow route is re-opened, and the restored ISS in an excited area will create a suction effect to some degree. As a result, the accumulated tracer molecules move both upstream and downstream and present as a slow-down of ISF flow. The first mechanism primarily depends upon the type of perineural net, wherein a more loose net results in an increase release of neurotransmitters into the space, and a reduced brain ISF flow^45^, whereas in the second mechanism, the reduction in flow speed is only related to a subpopulation of neural cells (i.e., astrocytes).

In addition to test these two hypotheses, it is desirable to identify the structural basis of the difference in flow and transport behavior between thalamus and caudate nucleus regions of the brain, and develop other approaches to control or regulate the transport of substances in the brain ISS. These studies will improve our understanding of basic brain functions and mechanisms and ultimately lead to better treatment of brain disorders via more effective drug delivery through the brain ISS.

## Materials and Methods

### Rats

Male adult Sprague-Dawley rats weighing 250–300 g were used. Rats were single housed under 12-hour light/dark cycles. Temperature (22 ± 1 °C) and humidity (60 ± 5%) were controlled. Rats were anesthetized via an intraperitoneal injection of sodium pentobarbital (50 mg/kg) and anesthesia was maintained with ~30 mg/kg/h sodium pentobarbital during the operation^46^,^47^. All experiments using rats were in accordance with the national guidelines for the use of experimental animals. The protocols were approved by the Ethics Committee of Peking University Health Center.

### Animal groups

Forty rats in total were randomly divided into five groups (n = 8 each group) (Table 1). In group Cc (caudate nucleus control), the tracer was injected into the center of the caudate nucleus, which is a non-pain-related region, and no painful stimulation was applied; in group Tc (thalamus control), the tracer was injected into the center of the thalamus, which is a pain-related region, and no painful stimulation was applied; in group Cs (caudate nucleus stimulation), the tracer was injected into the center of the caudate nucleus, which is a non-pain-related region, and the painful stimulation was applied with a current intensity of 3 mA; in group Ts (thalamus stimulation), the tracer was injected into the center of the thalamus, which is a pain-related region, and the painful stimulation was applied with a current intensity of 3 mA; and in group Tss (thalamus strong stimulation), the tracer was injected into the center of the thalamus, which is a pain-related region, and the stronger painful stimulations was applied with a current intensity of 5 mA.

### MRI scan protocols

**Figure 1:**
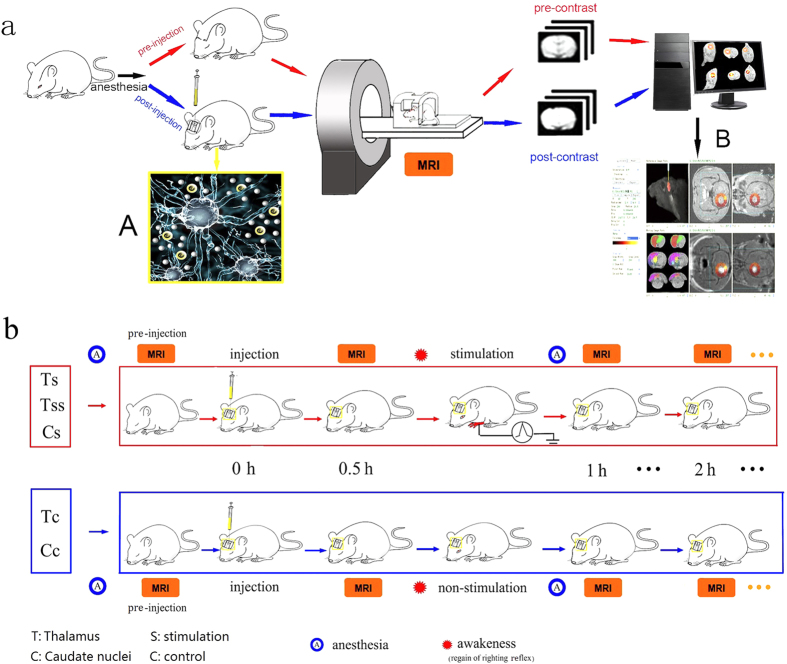
The experimental schematic diagram and operation procedure. (**a**) The schematic diagram for imaging the tracer’s transportation into the ISS or ISF flow process. MRI scans were performed before and after the injection of Gd-DTPA into the ISS of the rat brain under anesthesia (A). The MRI scans were performed at 30 min and each hour post-injection until no enhancement could be demonstrated on the subtracted MR images, which was obtained by a pixel-by-pixel co-registration and subtraction of the post-injection images from the pre-injection images (B). Developed software was used to perform the post-processing of the images. The transportation of the tracer in the ISS on the global, whole-brain scale can be shown three-dimensionally, and the flow or diffusion parameters can be derived from the time profile of the net enhancement on the subtracted images. (**b**) The procedure in *Ts* and *Tc* groups. *Ts*, *Tss* and *Cs* indicate the stimulation groups; *Tc* and *Cc* indicate the non-stimulation control groups. The anesthetized rat was scanned to acquire a basic image for further subtraction and was immobilized so that a small trephine hole could be made using a stereotaxic instrument (Lab Standard Stereotaxic-Single, Stoelting Co, Illinois, USA). 2 μl Gd-DTPA solution (10 mmol/L) was automatically infused into the brain ISS at the rate of 0.2 μl/min in all groups. The stereotactic coordinates for *T*. were set at the bregma: −3.0 mm, lateral: 2.0 mm, vertical: 6.0 mm and for *Cn*. at bregma: + 1.0 mm, lateral: 3.5 mm, vertical: 5.0 mm. The rats were then placed in the scanner to acquire the post-injection MR images at 0.5 h. When an MRI scan was applied at the 0.5-hour timepoint following injection, the painful stimulation was conducted in the *Ts* and *Tss* groups while a positive righting reflex was maintained to ensure the conscious state of the rats (see details in Fig. 4). No stimulation was conducted in the *Tc* or Cc groups. After the stimulation period, the rats were anesthetized and a series of MRI scans were performed.

**Figure 2:**
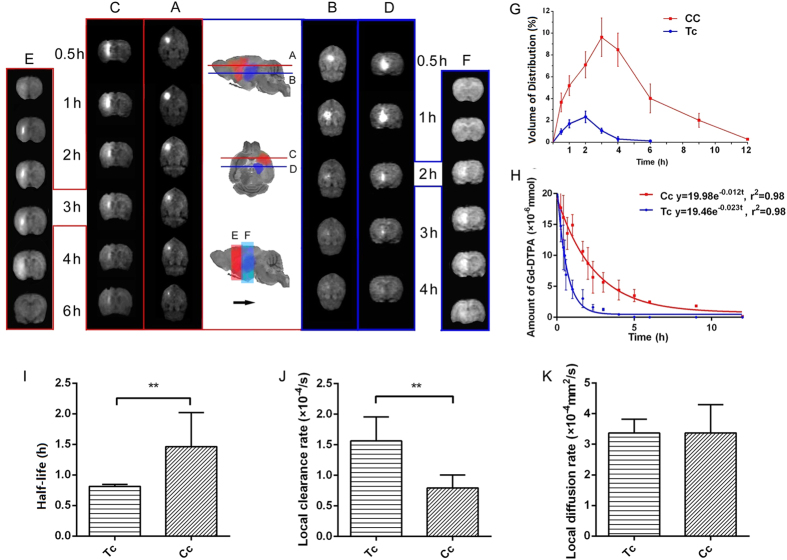
The dynamic distribution and clearance of the tracer in the ISS of Cc and Tc groups. *Cc*: Caudate nucleus group without painful electrical stimulation; *Tc*: thalamus group without painful electrical stimulation. Columns (**A,C)**, with red borders, indicate the orthogonal slices through the injection center in the *C*_*c*_ group. Columns (**B,D**), with blue borders, indicate the orthogonal slices through the injection center in the *T*_*c*_ group. The maximum distribution on the slice is shown in column (**E**) for the *C*_*c*_group and in column (**F**) for the *T*_*c*_ group. The line chart (**G**) profiles the tracer distribution in the *Cc* group (red) and *Tc* group (blue). The fitting curve (**H**) illustrates the exponential decay curves of the *Cc* group (red) and the *T*_*c*_ group (blue). The half-life of the *Tc* group was significantly shorter than that of the *Cc* group (**I**) (0.81 ± 0.03 hours in the *Tc* group, 1.46 ± 0.56 hours in the *Cc* group, *P* < 0.05, independent sample *t*-test). The local clearance in the *Tc* group was faster than in the *Cc* group (**J**) (1.56 ± 0.14 × 10-4/s in the *Tc* group, 0.79 ± 0.21 × 10-4/s in the *Cc* group, *P* < 0.05, independent sample *t*-test). No differences were observed for the diffusion parameters between the two groups (**K**) (Independent sample *t*-test, *P* > 0.05). The data are expressed as the mean ± standard deviation (SD).

**Figure 3:**
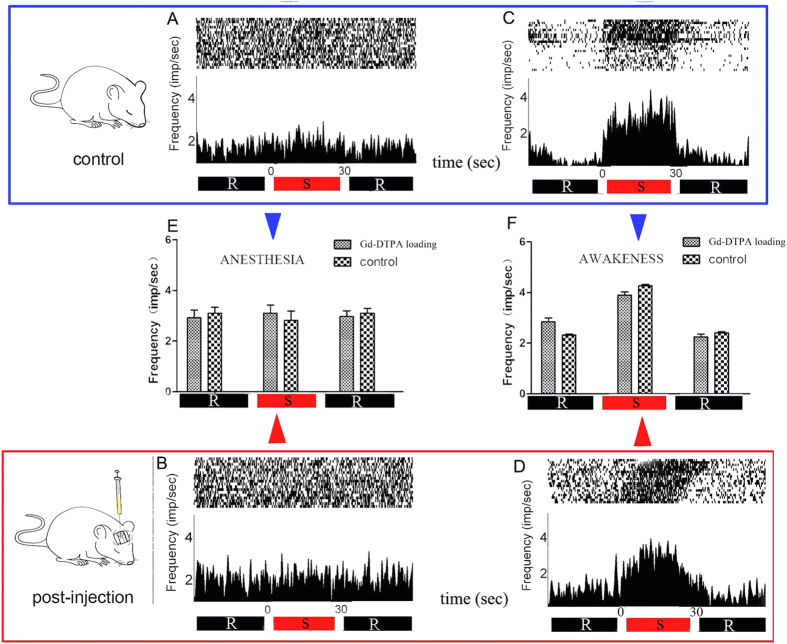
Rasters and perievent histograms in Ts-pre and Ts-post groups. The conduction process and neuronal activities evoked by painful stimulation were recorded in the thalamus and after the injection of a tracer (Gd-DTPA), which did not interfere with the thalamic recordings. (**A,B**) The neuronal responses of anesthetized rats during electrical stimulation before and after Gd-DTPA injection. (**C,D**): The neuronal responses during electrical stimulation in the awake state before and after Gd-DTPA injection. (**E,F**) The statistical analyses of (**A–D**). Similar neuronal spikes were detected before and after Gd-DTPA injection. Furthermore, awake rats were more sensitive than anesthetized rats to stimulation.

**Figure 4:**
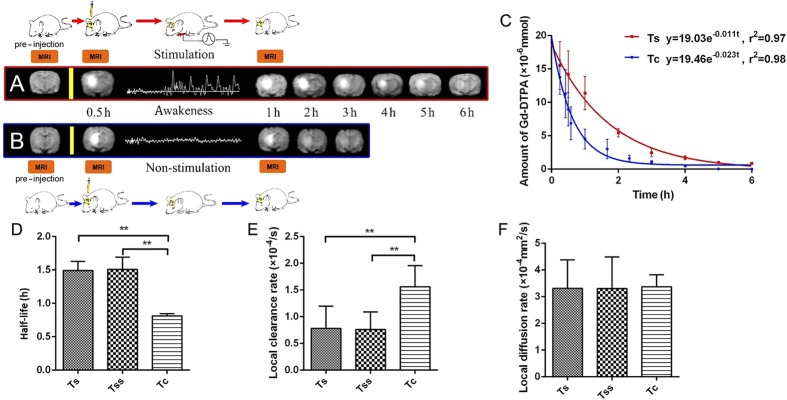
A significant reduction in the transportation velocity of the tracer in the ISS was observed in the pain-related thalamic region following painful stimulation. *Ts*: Thalamus group with painful electrical stimulation of 3 mA; *Tss*: thalamus group with painful electrical stimulation of 5 mA; *Tc*: thalamus group without painful electrical stimulation. Row (**A**) illustrates the slowing of the tracer clearance process in the *Ts* groups. The clearance process in the *Ts* group took nearly 6 hours, which corresponded to a t_½_ value of 1.49 ± 0.13 hours. Row (**B**) illustrates that the transportation in the *Tc* group was faster compared with the *Ts* group. The clearance of the tracer in the *Tc* took only 3 hours, which corresponded to a t_½_ value of 0.81 ± 0.03 hours. The chart (**C**) illustrates the exponential decay curves in the *Ts* group (red) and the *Tc* group (blue). A marked decrease in the transportation velocity of the tracer was found in the *Ts* and *Tss* groups, and the t_½_ values of the *Ts* and *Tss* groups were significantly longer compared with the *Tc* group (0.81 ± 0.03 hours in the *Tc* group, 1.49 ± 0.13 hours in the *Ts* group, 1.50 ± 0.18 hours in the *Tss* group; Tukey’s Multiple Comparison Test, *P* < 0.05) (**D**). No significant differences in the clearance or the flow speed were observed between the *Ts* and *Tss* groups (Tukey’s Multiple Comparison Test, *P* > 0.05). The local clearance rates in the *Ts* and *Tss* groups were less than those of the *Tc* group (**E**) (0.78 ± 0.41 × 10^–4^/s in the *Ts* group, 0.76 ± 0.33 × 10^–4^/s in the *Tss* groups, 1.55 ± 0.39 × 10^–4^/s in the *Tc* group; Tukey’s Multiple Comparison Test, *P* < 0.05). No differences were found in the diffusion parameters between all groups (**F**) (Tukey’s Multiple Comparison Test, *P* > 0.05). The data are expressed as the mean ± standard deviation (SD).

**Figure 5:**
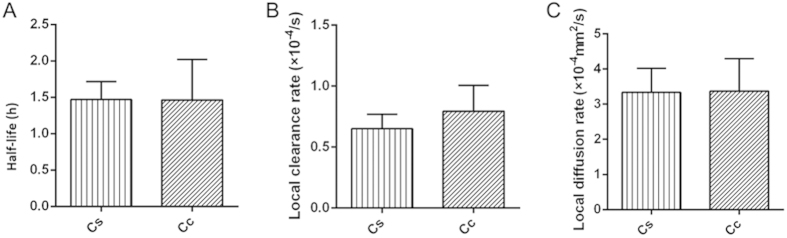
No significant differences were found in the half-life, local clearance rate and diffusion rate between the Cs group and Cc group. *Cs*: Caudate nucleus group with painful electrical stimulation; *Cc*: caudate nucleus group without painful electrical stimulation. (**A**) The half-life (*t*_*½*_) in the *Cs* group (1.47 ± 0.24 hours) did not differ from that of the *Cc* group (1.46 ± 0.56 hours) (independent sample *t-*test, *P > *0.05). (**B**) No significant difference was observed in the clearance rate constant *k’* between the *Cs* group (0.65 ± 0.11 × 10^–4^/s) and the *Cc* group (0.79 ± 0.21 × 10^–4^/s) (independent sample *t-*test, *P > *0.05). (**C**) No difference was observed for the diffusion parameters between the *Cs* group (3.37 ± 0.93 × 10^–4^ mm^2^/s) and the *Cc* group (3.33 ± 0.69 × 10^–4^ mm^2^/s) (independent sample *t*-test, *P* > 0.05). The data are expressed as the mean ± standard deviation (SD).

**Figure 6:**
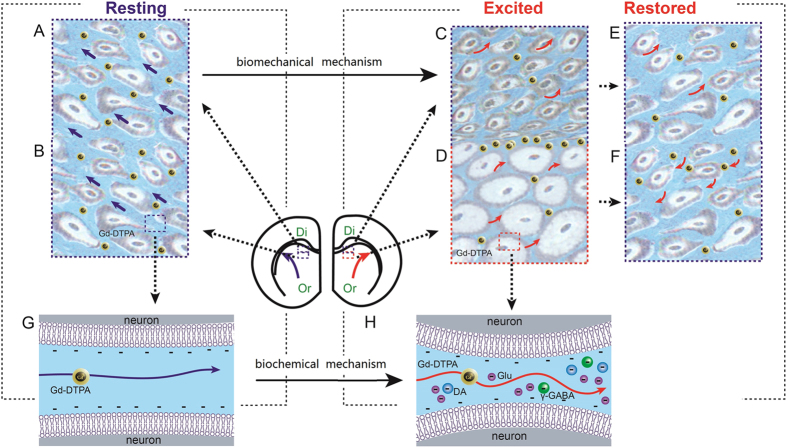
The mechanisms by which transportation is slowed following neuronal excitation. The biomechanical mechanism is illustrated in (**A–F**). (**A,B**) illustrate the transportation of tracer in ISS in a resting state; Or: original locations; Di: distal area. The blue arrows illustrate the direction in which the tracer is transported. (**C,D**) show the transportation of the tracer in the ISS after neuronal excitation; the astrocytes become swollen, the ISS is narrowed, the adjacent cells are flattened (and the ISS is even eliminated in some instances), and the ISF flow is reduced. (**E,F**) show the transportation of the tracer in the ISS when neuronal state is restored; distortions of the astrocytes and ISS are restored and the ISF flow returns to normal. The red arrows show the direction in which the tracer is transported. The chemical mechanism is illustrated in (**G,H**). (**G**) illustrates the transportation of a tracer in the ISS in a resting state. (**H**) illustrates the transportation of tracer in the ISS when neuronal excitation. Glu: glutamate; Da: dopamine.

The experimental procedure is illustrated in Fig. 1a. MRI examination was performed using a 3.0 T MRI scanner (Siemens; Germany) equipped with a dedicated coil. The anesthetized rats were placed in a prone position and were scanned with a 3D T1-weighted MP-RAGE sequence before and after the injection of the tracer. The acquisition parameters were as follows: echo time = 3.7 ms, repetition time = 1500 ms, flip angle = 12°, inversion time = 900 ms, field of view = 267 mm, voxel = 0.5 × 0.5 × 0.5 mm^3^, matrix = 512 × 96, number of averages = 2, phase-encoding steps = 96, and an acquisition time of 290 seconds. For each subject, scanning was performed before and after the introduction of Gd-DTPA. The scan time points were set as pre-injection, 15 minutes, 30 minutes and each hour post-injection until the “bright region” faded.

### Stereotaxic intracranial injections of Gd-DTPA

Gd-DTPA (Magnevist; Bayer Schering Pharma AG, Berlin, Germany) at 10 mmol/L was diluted with 154 mmol/L NaCl solution. To ensure the puncture position, MRI scanning was conducted before the injection and used to design the injection route and depth. Each rat was anesthetized and the core temperature was monitored with a rectal thermometer and maintained with a heating pad at (38 ± 0.5) °C. Additionally, the other physiological variables (such as blood pressure, heart rate and respiratory rate) were also monitored, which showed no significant differences between the groups (data not shown). The skin covering the calvaria was shaved and disinfected with iodized alcohol. An incision was made in the scalp along the sagittal suture from the interaural area to the interocular area. The membranes and muscle attachments were dissected free of the skull bone, and the bregma suture was exposed. The rat was immobilized in a stereotactic coordinate system (Lab Standard Stereotaxic-Single, Stoelting Co, Illinois, USA) and a small trephine hole was made according to the stereotactic coordinates of T (bregma: −3.0 mm, lateral: 2.0 mm, vertical: 6.0 mm) or *Cn* (bregma: + 1.0 mm, lateral: 3.5 mm, vertical: 5.0 mm). A 2 μl total volume of Gd-DTPA solution was delivered into the T area via a 10 μL microsyringe (Hamilton, Bonaduz AG, Switzerland) at a rate of 0.2 μl/min using an automated drug administration system (Harvard Apparatus, USA), followed by a 5-minute waiting period to avoid dorsal reflux along the needle track. The rat was then quickly placed in the scanner in a prone position for the post-injection scan according to the MRI scan protocols.

### Electrical stimulation

To perform the electrical forepaw stimulation, two needle electrodes were inserted under the skin in digits two and four of the right forepaw. Electrical pulse stimulation was given with a physiological and pharmacological experimental stimulator. Either 3 mA or 5 mA of current was applied. Each stimulation lasted for 15 s with a 5 s interval, and a rectangular pulse was continuously delivered for 10 min. The time point at which the stimulation was applied is illustrated in Fig. 1b. The rats were placed in the MRI scanner to acquire the MR images before and after the injection of Gd-DTPA. When an MRI scan was applied at 0.5 hour after the injection and the righting reflex was positive, the painful stimulation was conducted. After the stimulation period, the rats were anesthetized again, and a series of MRI scans were performed.

### Post-procedure calculations of physiological parameters

A MATLAB-based software was developed to co-register the MR images of individual rats before and after the injection. All images following the injection were automatically subjected to rigid transformation, similarity measurements, high-order interpolation, and an adaptive stochastic gradient descent optimization. These images were then subtracted from the pre-scanned images^48^. The acquired “bright areas,” which were obtained by establishing a seed point, and a threshold in the ROI were assumed to be related to the presence of the tracer. New sets of post-processing MR images in the horizontal, sagittal, and coronal planes with slice thicknesses of 1 mm were generated by the software. After the co-registration and subtraction process, the signal intensity within the target area of the processed MR images was measured and denoted by ΔSI, which was used in calculating the diffusion parameter in the rat brain interstitial space.

The brain tissue around the injection site appeared as a high-intensity spot on the MR image shortly after the injection of Gd-DTPA (Fig. 1). The enhancement of the MR signal intensity caused by Gd-DTPA was converted to its concentration using an empirical fitting process^17^. Therefore, both the flow and diffusion parameters of the brain ISF could be calculated based upon the obtained distribution of the tracer concentration. According to a modified diffusion equation, the equivalent diffusion coefficient D and clearance parameters k’ in each MRI pixel near the injection site could be derived. Here, *Vd*_*max*_ is defined as the ratio of the maximum volume distribution of traced brain ISF to the total rat brain volume, as measured using the above-described method^18^. Because the clearance of the tracer in the whole rat brain fit well onto a mono-exponential decay function, the clearance coefficient k and half-life period (t_½_) could be used to represent both the clearance rate and the transportation speed (Fig. 1). This situation arises because substance transportation in the ISS is attributed to diffusion and bulk flow; thus, the clearance rate can be used to depict the flow velocity of the tracer-injected ISF when the diffusion velocity is determined.

### Electrophysiological recording and data analysis

Rats were anesthetized using sodium pentobarbital (50 mg/kg, i.p.), as described previously, and were then transferred to the stereotaxic apparatus. Supplementary doses of sodium pentobarbital (~30 mg/kg/h) were given when necessary to maintain a sufficient level of anesthesia. A fitted rectangular window was made on the skull for microelectrode array implantation. The coordinates for the craniotomies were determined according to the atlas of Paxinos and Watson G^49^ as follows: (1) for Cn, bregma: + 1.0 mm, lateral: 3.5 mm, vertical: 5.0 mm; (2) for T, bregma: −3.0 mm, lateral: 2 mm, vertical: 6.0 mm. A 2 × 8 microarray was slowly lowered into the target areas. The microelectrode arrays were secured onto the cranium using dental cement and skull screws as anchors. Penicillin (80,000 U, i.m.) was administered to the rats for three days to prevent infection. The animals were housed individually after surgery. Two weeks after the initial microarray implantation surgery, the rats were anesthetized again, and a 2 μl total volume of Gd-DTPA was delivered into the T area via a preset micropipette. Then, the neuronal activity was recorded using a 64-channel single-unit recording system (Blackrock Microsystems, UT, USA) before and after the painful stimulation. The filtering wavelength and sampling rate for neuronal firing were set to 500 ~ 7500 Hz and 30 k/s, respectively. The sampling rate for the local field potential was 1 K/s with a filtering wavelength 0.5 ~ 500 Hz^50^.

The electrophysiological data were analyzed using Offline-sorter, NeuroExplorer and Matlab as follows: Offline-sorter was used to classify the recorded neuron and to remove the mechanical interference signals, and NeuroExplorer was used to investigate the interdependency between the neuronal firing and events. Neuronal activity that aligned with task events (e.g., stimulus presentation) was grouped into 50 ms bins, and spike density functions were generated by Gaussian smoothing of the resulting event-related histogram. The firing frequencies of characteristic neurons were analyzed statistically. After normalization, the frequency was superimposed for the exploration of the overall features.

### Statistical analysis

Statistical analyses were performed using the SPSS 19.0 software (SPSS Inc., Chicago, IL, USA). The data are expressed as the mean ± standard deviation (SD). To compare the half-life time (t_1/2_), clearance rate constant (k’), and the effective diffusion coefficient (D) between the Cc and Tc groups, independent sample *t*-tests were used. One-way analysis of variance (ANOVA) followed by Tukey’s multiple comparison test were used for the comparison among the Ts, Tss and Tc groups. An independent sample *t*-test was also used for the comparison between Cs and Cc groups. A *P*-value < 0.05 was considered statistically significant.

## Additional Information

**How to cite this article**: Shi, C. *et al.* Transportation in the Interstitial Space of the Brain Can Be Regulated by Neuronal Excitation. *Sci. Rep.* **5**, 17673; doi: 10.1038/srep17673 (2015).

## Figures and Tables

**Table 1:** Summary of the experimental groups and related items.

Group	Observed area	Pain-related	Painful stimulation and intensity
Cc	caudate nucleus	−	−/0 mA
Tc	thalamus	+	−/0 mA
Cs	caudate nucleus	−	+/3 mA
Ts	thalamus	+	+/3 mA
Tss	thalamus	+	+/5 mA
